# Optimal Timing of Anticoagulation Initiation in Stroke Patients With Atrial Fibrillation: A Systematic Review

**DOI:** 10.7759/cureus.88296

**Published:** 2025-07-19

**Authors:** Shaima Yasmeen Tariq, Ali Raza Ahmad

**Affiliations:** 1 Cardiology, Sahiwal Teaching Hospital, Sahiwal, PAK; 2 Hematology and Oncology, Hameed Latif Hospital Lahore, Lahore, PAK

**Keywords:** anticoagulation, atrial fibrillation, intracranial hemorrhage, ischemic stroke, stroke, timing, warfarin

## Abstract

This systematic review evaluates the optimal timing of anticoagulation in atrial fibrillation patients with acute ischemic stroke or transient ischemic attack. A comprehensive search across PubMed (94 records), Web of Science (121 records), Scopus (156 records), and the Cochrane Library (83 records) yielded 454 records. After screening 324 records and assessing 44 full texts, 13 studies were included: five randomized controlled trials and eight cohort studies, excluding those without comparisons of early versus delayed anticoagulation. The review analyzes studies comparing early and delayed initiation of oral anticoagulants (primarily direct oral anticoagulants (DOACs) and warfarin), focusing on outcomes such as recurrent ischemic stroke, symptomatic intracranial hemorrhage (sICH), and composite endpoints including recurrent stroke, sICH, systemic embolism, or mortality. Findings suggest that early anticoagulation, especially with DOACs, is safe and may reduce recurrent events without increasing the risk of sICH, particularly in cases with mild to moderate severity. However, varying definitions of timing, study designs, and limited data on severe strokes or in low-resource settings highlight research gaps. Future studies should investigate long-term outcomes and diverse populations to refine guidelines, particularly in resource-constrained environments.

## Introduction and background

Stroke is a leading cause of disability and mortality globally, imposing a significant burden on healthcare systems and patients. In Pakistan, an estimated 350,000 new stroke cases occur annually, reflecting a significant national health burden. Globally, around 12.2 million new stroke cases were reported in 2019, underscoring the urgent need for effective prevention and management strategies [1-3]. Atrial fibrillation (AF) is a significant risk factor for stroke, increasing the risk by fivefold, and is also linked to coronary artery disease, myocardial infarction, heart failure, dementia, and increased mortality [4]. AF is responsible for 15-20% of all strokes and significantly contributes to stroke-related morbidity and mortality [5]. Effective anticoagulation therapy is essential for stroke prevention in patients with AF, as it reduces thrombus formation and subsequent embolization to the brain [6,7]. However, the timing of anticoagulation initiation remains a complex clinical decision, as it involves balancing the risks of hemorrhagic transformation (conversion of ischemic brain tissue into a hemorrhagic area due to vascular fragility and reperfusion) with the potential for recurrent ischemic events [8,9]. Early anticoagulation in patients with large infarcts or vulnerable cerebral vasculature may increase the risk of symptomatic intracranial hemorrhage (sICH), while delaying anticoagulation could increase the risk of recurrent ischemic strokes, which may worsen neurological function and disability. Despite the clinical importance of timely anticoagulation initiation, the optimal timing for starting therapy in AF-related stroke has been a subject of ongoing debate. Different studies have yielded mixed results, with no consensus regarding the safest and most effective timeframe. Early initiation may benefit patients by preventing recurrent strokes, but it may also increase the risk of bleeding complications. Conversely, delayed anticoagulation might reduce hemorrhagic risk but may also leave patients vulnerable to ischemic events.

This review aims to evaluate existing evidence on the optimal timing for initiating anticoagulation in stroke patients with AF. While current guidelines, including those from the American Heart Association/American Stroke Association, recommend tailoring initiation based on stroke severity, they do not provide clear guidance on the distinction between early and delayed anticoagulation. To address this gap, the review systematically examines studies comparing the outcomes of different initiation timings, focusing on recurrent ischemic stroke, hemorrhagic complications, and survival. The results are intended to support clinical decision-making and guide anticoagulation strategies, particularly in settings with limited access to specialized stroke services, such as Pakistan, where resource constraints may influence treatment practices and outcomes.

## Review

Methodology

This systematic review was conducted in 2025, following the Preferred Reporting Items for Systematic Reviews and Meta-Analyses (PRISMA) framework. A literature search was conducted using PubMed, Web of Science, Scopus, and the Cochrane Library, yielding 454 records (94, 121, 156, and 83, respectively) from 2000 to 2025. This timeframe aligns with the introduction of direct oral anticoagulants (DOACs), which occurred around 2000, and is consistent with modern anticoagulation practices. The search used Boolean operators (AND, OR) and MeSH terms (e.g., "atrial fibrillation," "stroke," "anticoagulation," "timing") with synonyms (e.g., "early," "late," "DOAC," "warfarin") to identify studies on anticoagulation timing in adults with AF and acute ischemic stroke (AIS) or transient ischemic attack (TIA). After removing 130 duplicates via EndNote (Clarivate, London, UK) and manual checks, 324 records were screened. Titles and findings from 13 studies, including randomized controlled trials (RCTs) and prospective and retrospective cohort studies, explore the timing of anticoagulation initiation in patients with AF and AIS or TIA [6,10-21]. These studies compare early versus delayed anticoagulation, assessing outcomes such as recurrent ischemic stroke, sICH, systemic embolism, cognitive and functional recovery, and mortality. Results indicate that early anticoagulation, particularly with DOACs, appears safe and potentially beneficial across various stroke severities and initiation timeframes. Abstracts were reviewed by two independent reviewers, excluding 218 irrelevant records, leaving 106 articles for full-text retrieval. Of these, 62 could not be obtained despite attempts via interlibrary loans and author contact, potentially introducing selection bias; however, the included studies were representative of key outcomes. Forty-four articles were assessed, and 31 were excluded (12 lacked early vs. delayed comparisons, eight addressed non-AF strokes, five used only parenteral anticoagulants, three omitted clinical outcomes, and three were narrative reviews). Thirteen studies (five RCTs and eight observational studies) were included in the qualitative synthesis, comparing early (≤24 hours to ≤7 days) versus delayed (>24 hours to >14 days) OAC initiation in AF-related stroke. The review was not registered in PROSPERO due to resource constraints; however, transparency was maintained through the use of a PRISMA flow diagram.

The target population consisted of adults aged 18 years or older diagnosed with AF and experiencing AIS or TIA, requiring anticoagulation for secondary stroke prevention. The intervention involved initiating oral anticoagulants (OACs), such as DOACs or warfarin, either early (ranging from ≤24 hours to ≤7 days across studies) or delayed (from >24 hours to >14 days, depending on the study design). Eligible study types included RCTs and prospective or retrospective cohort studies. Exclusion criteria covered pediatric patients, non-AF strokes, exclusive use of parenteral agents, lack of comparative arms, or focus on post-ICH populations without AIS/TIA relevance, except where AF-related outcomes were reported (e.g., Pennlert et al. [10], included for its AF anticoagulation data).

**Figure 1:**
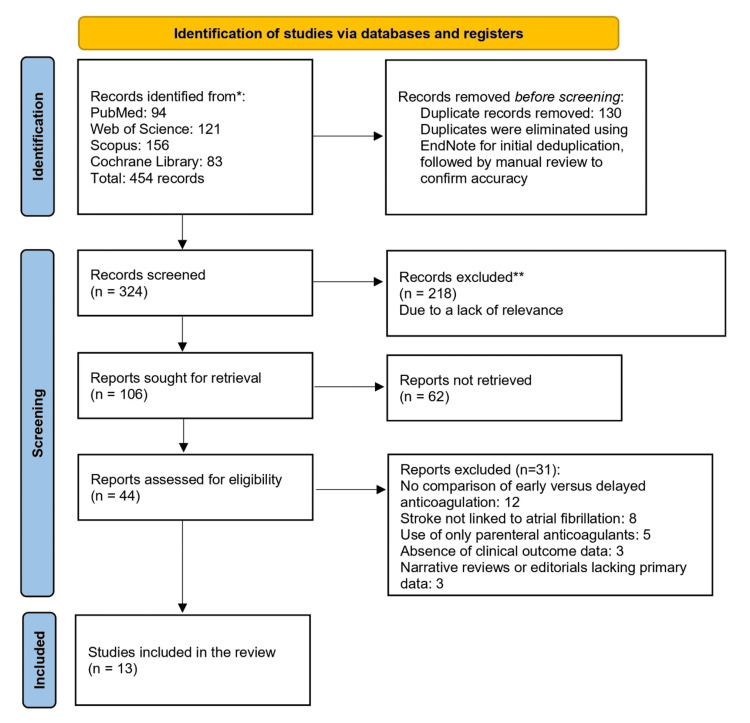
PRISMA flow diagram of the study selection process PRISMA 2020 flow diagram for new systematic reviews, which included searches of databases and registers only. * Consider, if feasible to do so, reporting the number of records identified from each database or register searched (rather than the total number across all databases/registers). ** If automation tools were used, indicate how many records were excluded by a human and how many were excluded by automation tools. PRISMA: Preferred Reporting Items for Systematic Reviews and Meta-Analyses

The primary outcomes assessed were recurrent ischemic stroke, sICH, and composite endpoints comprising any combination of recurrent stroke, sICH, systemic embolism, or all-cause mortality. Data extraction was conducted using a standardized template, capturing information on study design, sample size, details of the anticoagulation intervention, outcome measures, and key findings. Following the removal of duplicates, two independent reviewers screened titles and abstracts for relevance. Full-text articles of potentially eligible studies were then assessed. Any disagreements between reviewers were resolved through discussion with a third reviewer to achieve consensus. A meta-analysis was not conducted due to significant heterogeneity in study designs, definitions of early and delayed anticoagulation timing, and variations in reported outcome measures across the included studies, which precluded the estimation of a meaningful pooled effect. The study selection process is illustrated in Figure 1, which presents the PRISMA flow diagram outlining the number of records identified, screened, excluded, and ultimately included in the review.

Results

Findings from 13 studies, including RCTs and prospective and retrospective cohort studies, examine the timing of anticoagulation initiation in patients with AF and AIS or TIA [6,10-21]. These studies compare early versus delayed anticoagulation, assessing outcomes such as recurrent ischemic stroke, sICH, systemic embolism, cognitive and functional recovery, and mortality. Results indicate that early anticoagulation, particularly with DOACs, appears safe and potentially beneficial across various stroke severities and initiation timeframes, as summarized in Table 1.

**Table 1:** Summary of studies evaluating the timing of anticoagulation initiation in patients with AF and AIS or TIA ADL: activities of daily living, AF: atrial fibrillation, aHR: adjusted hazard ratio, AIS: acute ischemic stroke, CI: confidence interval, DOAC: direct oral anticoagulant, ECH: extracranial hemorrhage, ICH: intracranial hemorrhage, MoCA: Montreal Cognitive Assessment, NIHSS: National Institutes of Health Stroke Scale, NOAC: non-vitamin K antagonist oral anticoagulant, OAC: oral anticoagulant, OAT: oral anticoagulant therapy, OR: odds ratio, RD: risk difference, sICH: symptomatic intracranial hemorrhage, TIA: transient ischemic attack

Study	Study population	Intervention details	Measured outcomes	Main results
De Marchis et al., 2022 [6]	2,550 patients with AIS and non-valvular AF	DOACs initiated early (≤median, n=1,362) or late (>median, n=1,188) within 30 days	Recurrent AIS and ICH within 30 days	No significant differences in recurrent AIS (1.7% early vs. 1.2% late, aHR 1.2, 95% CI 0.5-2.9) or ICH (0.1% vs. 0.3%, aHR 6.0, 95% CI 0.6-56.3). Early DOAC initiation is considered safe.
Deguchi et al., 2017 [11]	300 patients with AIS and non-valvular AF	DOACs (median 3 days, n=186) vs. warfarin (median 7 days, n=114)	Timing of OAC initiation by stroke severity and hemorrhagic events	DOACs initiated earlier than warfarin (p<0.001): 2 days for mild, 7 days for moderate, 11 days for severe strokes. No ICH in DOAC group; 3 hemorrhagic events in warfarin group.
Elsayed et al., 2025 [12]	300 patients with AIS and confirmed AF	Early anticoagulation (≤24h, n=150) vs. late (>24h, n=150)	Cognitive (MoCA) and functional (ADL) outcomes at 6 months	Early anticoagulation resulted in better cognitive outcomes (MoCA: 24.5 ± 3.5 vs. 21.8 ± 4.0, p<0.01) and improved ADL scores, indicating enhanced functional recovery.
Fischer et al., 2023 [13]	2,013 patients with AIS (37% minor, 40% moderate, 23% major stroke)	Early anticoagulation (within 48h for minor/moderate, day 6-7 for major, n=1,006) vs. later (day 3-4 minor, 6-7 moderate, 12-14 major, n=1,007)	Recurrent ischemic stroke, sICH, and death at 30 and 90 days	Early anticoagulation reduced the risk of recurrent stroke (1.4% vs. 2.5%, OR 0.57, 95% CI 0.29-1.07) and composite events (2.9% vs. 4.1%, risk difference -1.18%, 95% CI -2.84 to 0.47) at 30 days. sICH rates were equal (0.2% in both).
Goeldlin et al., 2024 [14]	1,962 patients with AIS, categorized by stroke severity (minor, moderate, major)	Early DOAC initiation (≤48h for minor/moderate, day 6-7 for major, n=978) vs. late (day 3-4 minor, 6-7 moderate, 12-14 major, n=984)	Composite of recurrent ischemic stroke, sICH, extracranial bleeding, systemic embolism, or vascular death at 30 days	No significant differences in composite outcome by stroke severity: minor (2.7% vs. 3.0%, OR 0.89, 95% CI 0.38-2.10), moderate (2.8% vs. 3.6%, OR 0.80, 95% CI 0.35-1.74), major (3.7% vs. 7.0%, OR 0.52, 95% CI 0.21-1.18).
Kimura et al., 2022 [15]	1,797 patients with TIA or AIS, stratified by stroke severity (mild, moderate, severe)	Early DOAC initiation (≤1-4 days, n=785) vs. late (>1-4 days, n=1,012)	Composite of recurrent stroke, systemic embolism, and major bleeding within 90 days	Early DOAC use was associated with a reduced risk of stroke/systemic embolism (1.9% vs. 3.9%, aHR 0.50, 95% CI 0.27-0.89) and ischemic stroke (1.7% vs. 3.2%, aHR 0.54, 95% CI 0.27-0.999). Bleeding rates were similar (0.8% vs. 1.0%).
Mizoguchi et al., 2020 [16]	499 patients with AIS or TIA and non-valvular AF	Early DOAC initiation (≤3 days, n=223) vs. late (≥4 days, n=276)	Stroke, systemic embolism, major bleeding, and death at 3 months and 2 years	No significant differences in stroke/embolism (HR 0.86, 95% CI 0.47-1.57), bleeding (HR 1.39, 95% CI 0.42-4.60), or death (HR 0.61, 95% CI 0.28-1.33) at 3 months or 2 years.
Oldgren et al., 2022 [17]	888 patients with AIS and AF	Early NOAC initiation (≤4 days, n=450) vs. delayed (5-10 days, n=438)	Composite of recurrent ischemic stroke, symptomatic ICH, and all-cause mortality at 90 days	Early NOAC initiation was non-inferior (6.89% vs. 8.68%, risk difference -1.79%, 95% CI -5.31 to 1.74). Ischemic stroke rates: 3.11% vs. 4.57%; no sICH occurred in either group.
Paciaroni et al., 2020 [18]	2,470 patients with AIS (19.1% posterior, 80.9% anterior) and AF	OAT within 2 days or between 3-7 days	Composite of stroke recurrence, TIA, systemic embolism, and symptomatic bleeding within 90 days	No difference in composite outcome by stroke location: within 2 days (5.3% posterior vs. 4.3% anterior, OR 1.07, 95% CI 0.39-2.94); 3-7 days (2.9% vs. 5.3%, OR 0.54, 95% CI 0.16-1.80).
Pennlert et al., 2017 [10]	2,619 survivors of ICH with AF	Anticoagulation started 7-8 weeks post-ICH vs. no anticoagulation	Vascular death or nonfatal stroke within 3 years	Anticoagulation at 7-8 weeks reduced vascular death/stroke risk (17.0% vs. 28.6% for women, 14.3% vs. 23.6% for men, p<0.05) without significantly increasing severe bleeding.
Werring et al., 2024 [19]	3,621 patients with AIS and AF	Early DOAC initiation (≤4 days, n=1,814) vs. delayed (7-14 days, n=1,807)	Composite of recurrent ischemic stroke, sICH, unclassifiable stroke, and systemic embolism at 90 days	No difference in composite outcome (3.3% in both groups, adjusted risk difference 0.000, 95% CI -0.011 to 0.012). sICH rates were similar (0.6% early vs. 0.7% late). Early DOAC initiation was non-inferior.
Wilson et al., 2019 [20]	1,355 patients with AIS or TIA and AF	Early OAC initiation (0-4 days, n=358) vs. late (≥5 days or never, n=997)	Composite of TIA, ischemic stroke, ICH, or death within 90 days	No significant difference in composite outcome (2% early vs. 5% late, adjusted OR 1.17, 95% CI 0.48-2.84). Early OAC initiation showed no increased risk.
Yaghi et al., 2020 [21]	1,289 patients with AIS and AF, selected from 2,084 screened	Anticoagulation started early (0-3 days, n=617), intermediate (4-14 days, n=535), or late (>14 days, n=137)	Composite of ischemic events, sICH, and ECH	No notable differences in composite endpoint rates (10.3% early, 9.7% intermediate, 10.2% late; p=0.933). No significant reduction in sICH (OR 1.49, 95% CI 0.50-4.43, 4-14 vs. 0-3 days) or ischemic events (OR 0.76, 95% CI 0.36-1.62, 4-14 vs. >14 days).

Discussion

The timing of anticoagulation initiation in patients with AF following AIS or TIA involves balancing the risks of recurrent thromboembolism against hemorrhagic complications, particularly sICH. This systematic review synthesizes evidence from 13 studies, indicating that early anticoagulation, typically within one to four days for minor to moderate strokes (National Institutes of Health Stroke Scale (NIHSS) <15) and six to seven days for severe strokes (NIHSS ≥15), is generally safe and may reduce recurrent ischemic events without significantly increasing sICH risk, particularly with DOACs [7,10,18,22].

The reviewed studies collectively suggest that early anticoagulation, especially with DOACs, is safe and potentially beneficial across a spectrum of stroke severities. Fischer et al. found that early anticoagulation (within 48 hours for minor/moderate strokes, six to seven days for major strokes) reduced recurrent ischemic stroke rates at 30 days (1.4% vs. 2.5%, OR 0.57, 95% CI 0.29-1.07) compared to delayed initiation, with equivalent sICH rates (0.2%) [13]. Similarly, Kimura et al. reported that early DOAC initiation (≤1-4 days) reduced the composite of recurrent stroke and systemic embolism (1.9% vs. 3.9%, aHR 0.50, 95% CI 0.27-0.89) without increasing bleeding risk (0.8% vs. 1.0%) [15]. These findings align with Oldgren et al., who demonstrated non-inferiority of early DOAC initiation (≤4 days) compared to delayed initiation (5-10 days) for composite outcomes (6.89% vs. 8.68%, risk difference -1.79%, 95% CI -5.31 to 1.74), with no sICH in either group [17]. Werring et al. (2024) further supported this finding, noting no difference in composite outcomes between early (≤4 days) and delayed (7-14 days) DOAC initiation (3.3% in both groups) [19].

In contrast, Yaghi et al. found no significant differences in composite vascular events across early (0-3 days), intermediate (4-14 days), and late (>14 days) anticoagulation groups (10.3% early, 9.7% intermediate, 10.2% late; p=0.933). This discrepancy stems from differences in study populations (inclusion criteria or baseline risk profiles); for example, "early" ranges from ≤24 hours to ≤4 days, and "late" ranges from >24 hours to >14 days, complicating direct comparisons. Low event rates and wide confidence intervals, as seen in Yaghi et al. (OR 1.49, 95% CI 0.50-4.43 for sICH), may obscure true differences [21].

Stroke severity, as assessed by the NIHSS, has a significant influence on the timing of anticoagulation. For minor to moderate strokes, early initiation (one to four days) is well-supported. Deguchi et al. reported median initiation times of two days for mild strokes, seven days for moderate strokes, and 11 days for severe strokes, reflecting caution in severe cases due to hemorrhagic transformation risks [11]. However, Goeldlin et al. suggested that early DOAC initiation (six to seven days) is feasible even in major strokes, reducing composite events (3.7% vs. 7.0%, OR 0.52, 95% CI 0.21-1.18), although this difference was not statistically significant due to the low event rates. This challenges traditional recommendations, such as delaying anticoagulation for 12-14 days in severe strokes, suggesting that earlier initiation may be safe with careful patient selection, guided by neuroimaging to assess infarct size and hemorrhagic risk [14]. Paciaroni et al. found no significant differences in composite outcomes based on stroke location (posterior vs. anterior), suggesting that severity, rather than location, influences timing decisions. This shift toward earlier initiation, even in severe strokes, reflects evolving evidence but requires validation in diverse populations and settings with limited access to imaging [18].

DOACs consistently demonstrate a superior safety profile over warfarin in the early post-stroke period. Deguchi et al. reported no hemorrhagic events with DOACs (median initiation three days) compared to three with warfarin (median seven days), attributed to DOACs' rapid onset, shorter half-life, and reduced need for bridging therapy with heparin, a known bleeding risk factor [11]. Mizoguchi et al. found no significant differences in outcomes between early (≤3 days) and late (≥4 days) DOAC initiation, thereby reinforcing the safety of DOACs across various timing windows. Warfarin's delayed therapeutic effect and need for International Normalized Ratio (INR) monitoring make it less suitable for early initiation, particularly in moderate to severe strokes, where rapid anticoagulation is critical [16].

Early anticoagulation may confer additional benefits in preserving functional and cognitive outcomes. Elsayed et al. found that anticoagulation within 24 hours improved cognitive performance (Montreal Cognitive Assessment: 24.5 ± 3.5 vs. 21.8 ± 4.0, p<0.01) and activities of daily living scores at six months. This may be due to the prevention of silent microemboli, which can cause cumulative neuronal damage, and preservation of the ischemic penumbra through sustained cerebral perfusion, reducing secondary injury [12]. These benefits are particularly relevant in resource-limited settings like Pakistan, where rehabilitation services are scarce, and optimizing early intervention can reduce long-term disability. Pennlert et al. studied anticoagulation timing in 2,619 post-ICH patients with AF, finding that initiation at seven to eight weeks reduced vascular death or nonfatal stroke (17.0% vs. 28.6% for women, 14.3% vs. 23.6% for men, p<0.05) without increasing bleeding risk. While these findings highlight the long-term benefits of anticoagulation in AF patients with prior cerebrovascular events, extrapolating them to AIS/TIA populations is limited. ICH patients face distinct risks, such as a higher baseline bleeding propensity, which may not apply to AIS/TIA cases. Thus, while Pennlert et al.'s results support the benefits of anticoagulation, their relevance to AIS/TIA timing decisions requires cautious interpretation [10].

Current guidelines, including those from the American Heart Association, recommend delaying anticoagulation for 7-14 days in severe strokes to minimize hemorrhagic complications, while allowing earlier initiation within 2-4 days for minor strokes. Evidence from Fischer et al., Goeldlin et al., and Kimura et al. suggests that initiating DOACs as early as one to four days for minor to moderate strokes and six to seven days for severe strokes may be both safe and effective. These findings, when interpreted in conjunction with individualized factors such as infarct size, renal function, neuroimaging features like hemorrhagic transformation or cerebral microbleeds, and concurrent antiplatelet therapy, suggest that existing timing strategies may require refinement [13-15,21,23].

In low-resource settings, such as Pakistan, implementing early anticoagulation faces significant barriers. Limited access to neuroimaging (CT/MRI) hinders accurate assessment of hemorrhagic risk, often necessitating reliance on non-contrast CT and clinical judgment. DOACs, despite their safety and efficacy, are cost-prohibitive, and warfarin's INR monitoring is logistically challenging in areas with limited laboratory infrastructure. Elsayed et al.'s findings on the benefits of early anticoagulation are compelling, but their implementation is challenging without rapid diagnostic evaluation and follow-up [12]. Health system interventions, such as subsidizing DOAC costs, improving access to basic imaging, or training providers to use risk scores, could standardize decision-making. Patient education is also critical, as DOACs require strict adherence to dosing schedules.

The convergence of evidence supports early DOAC initiation as a safe and effective strategy for AF-related AIS/TIA, particularly for minor to moderate strokes. However, discrepancies, such as those in Yaghi et al. [21], underscore the need for standardized timing definitions and larger, more diverse trials. The potential for early anticoagulation to improve cognitive and functional outcomes warrants further exploration, particularly in resource-limited settings where disability imposes a significant burden. Future research should focus on long-term outcomes, severe stroke populations, and low-resource contexts, using advanced imaging and risk stratification to refine timing strategies.

Limitations

Several factors limit the interpretation of findings on anticoagulation timing in AF-related AIS. Variability in study designs, definitions of "early" (≤24 hours to ≤4 days) and "late" (3-14 days or later), and composite endpoints (ischemic stroke, sICH, systemic embolism, death) hinder direct comparisons. Observational studies risk selection bias, while RCTs may exclude high-risk patients, and data on severe strokes are limited for very early anticoagulation (≤48 hours). Differences in anticoagulant types, predominantly focusing on short-term outcomes (30-90 days), and a focus on high-income settings may not accurately reflect low-resource contexts, such as Pakistan, where imaging and follow-up are scarce. Potential publication bias may also lead to an overrepresentation of positive findings.

## Conclusions

This systematic review of 13 studies suggests that early anticoagulation (≤24 hours to ≤4 days) in AF-related AIS or transient ischemic attack is generally safe, particularly with DOACs, and may reduce recurrent ischemic events without significantly increasing the risk of sICH in mild to moderate strokes. However, uncertainty remains for severe strokes due to limited data, and high-risk populations are underrepresented. Variability in timing definitions and outcome measures, along with potential publication bias, limits the comparability of results across studies. In low-resource settings, such as Pakistan, where advanced imaging may be unavailable, non-contrast CT and clinical judgment can help guide the timing of anticoagulation. These findings support consideration of earlier DOAC initiation in selected patients and highlight the need for future research to standardize timing criteria, assess long-term outcomes, and include diverse and resource-constrained populations.
